# The Val34Met, Thr164Ile and Ser220Cys Polymorphisms of the β2-Adrenergic Receptor and Their Consequences on the Receptor Conformational Features: A Molecular Dynamics Simulation Study

**DOI:** 10.3390/ijms23105449

**Published:** 2022-05-13

**Authors:** Aneta Archala, Wojciech Plazinski, Anita Plazinska

**Affiliations:** 1 Department of Biopharmacy, Medical University of Lublin, Chodzki 4a, 20-093 Lublin, Poland; anetaarchala@umlub.pl (A.A.); wojtek_plazinski@o2.pl (W.P.); 2 Jerzy Haber Institute of Catalysis and Surface Chemistry, Polish Academy of Sciences, Niezapominajek 8, 30-239 Krakow, Poland

**Keywords:** GPCR, β2-adrenergic receptor, polymorphism, molecular dynamics

## Abstract

The gene encoding the β2-adrenergic receptor (β2-AR) is polymorphic, which results in possible differences in a primary structure of this protein. It has been shown that certain types of polymorphisms are correlated with some clinical features of asthma, including airways reactivity, whereas the influence of other is not yet understood. Among polymorphisms affecting amino acids at positions 16, 27, 34, 164 and 220, the latter three are present in the crystal structure of β2-AR, which facilitates studying them by means of molecular dynamics simulations. The current study was focused on investigating to what extent the three polymorphisms of β2-AR (i.e., Val34Met, Thr164Ile and Ser220Cys) affect the interaction of β2-AR with its natural molecular environment which includes: lipid bilayer (in the case of all three polymorphs) and Gs protein (which participates in β2-AR-mediated signaling; in the case of Ser220Cys). We have designed and carried out a series of molecular dynamics simulations at different level of resolution (i.e., either coarse-grained or atomistic simulations), accompanied by thermodynamic integration protocol, in order to identify potential polymorphism-induced alterations in structural, conformational or energetic features of β2-AR. The results indicate the lack of significant differences in the case of energies involved in the β2-AR-lipid bilayer interactions. Some differences have been observed when considering the polymorphism-induced alterations in β2-AR-Gs protein binding, but their magnitude is also negligible in relation to the absolute free energy difference correlated with the β2-AR-Gs affinity. The Val34Met and Thr164Ile polymorphisms are weakly correlated with alteration of the conformational features of the receptor around polymorphic sites. On the contrary, it has been concluded that the Ser220Cys polymorphism is correlated with several structural alterations located in the intracellular region of β2-AR, which can induce G-protein binding and, subsequently, the polymorphism-correlated therapeutic responses. More precisely, these alterations involve vicinity of intracellular loops and, in part, are the direct consequence of disturbed interactions of Ser/Cys220 sidechain within 5th transmembrane domain. Structurally, the dynamic structure exhibited by the β2-AR^Ser220^ polymorph is closer to the Gs-compatible structure of β2-AR.

## 1. Introduction

The β2 adrenergic receptor (β2-AR) is a type of G protein-coupled receptor (GPCR), which represent the largest class of membrane receptors, with more than 800 members identified in the human genome [1]. GPCRs can bind a wide diversity of ligands that regulate most physiological processes and approximately 35% of approved drugs target GPCRs. As such, GPCRs constitute the largest family of proteins targeted by approved drugs [2]. β2-AR plays a key role in cardiovascular and pulmonary physiology [3,4,5]. The agonist molecule binding by β2-AR causes a sympathetic nervous system response, resulting in increased a heart rate, pupil dilation, rapid energy mobilization, redirection of blood to skeletal muscles and bronchial smooth muscle relaxation [1].

The β2 adrenoceptor was the first ligand-binding GPCR to be purified from cell membranes; the first to be cloned and sequenced [6] and it provided the first high-resolution crystal structures [7,8,9] and was the first receptor to be resolved in complex with its Gs protein (stimulatory G protein) [10,11]. Heterotrimeric G proteins located within the cell are activated by GPCRs. The stimulatory G protein (Gs) activates adenylate cyclase to increase intracellular cAMP (cyclic adenosine monophosphate), and it distributes ubiquitously in the tissues composing a housekeeping signaling pathway.

Despite these achievements, it remains unclear why the therapeutic response of several drugs is altered by polymorphic variations in the primary structure of the target GPCR. Single nucleotide polymorphism (SNP) is the phenomenon of DNA sequence variation, which consists in changing a single nucleotide between individuals of a given species, occurring in more than 1% of the population. Single nucleotide polymorphisms can be found in the coding sequences of genes, the noncoding regions of genes or in the intergenic regions. A single nucleotide polymorphism in the coding sequence of a gene does not necessarily lead to a change in the amino acid sequence of the protein, due to the degeneracy of the genetic code. Therefore, SNPs are classified as synonymous or nonsynonymous (when the resulting protein sequence has a changed amino acid due to polymorphism).

The primary sequence of β2-AR differs in humans. At least 49 SNPs have been identified in the β2-AR. Of these, 31 are >3% in Caucasians and African Americans. SNPs are organized into combinations that are inherited together into 24 haplotypes, each with a frequency of >1% [12]. The presence of polymorphic variants varies among people of different ethnic origins. Large-scale whole genome sequencing projects provide insight into the burden of rare polymorphs in different populations through multi-center studies that include the 1000 Genomes Project Consortium; the National Institutes of Health (NIH)/National Heart, Lung, and Blood Institute (NHLBI) GO Exome Sequencing Project [13].

Several SNPs have been described for β2-AR that are associated with disease susceptibility and a differential therapeutic response [14]. Clinical studies show a correlation of polymorphism with an increased risk of many chronic diseases (e.g., heart failure, hypertension, obesity, concomitant asthma and polycystic ovarian syndrome), a varying response to some drugs and a more intensive course of disease as well as a faster development of tolerance to the medication. Two common nonsynonymous SNPs have been the most extensively studied, namely Arg16Gly and Gln27Glu [15,16]. Their occurrence results in receptor function change, different ligand binding and impaired signal transmission. The Arg16Gly and Gln27Glu polymorphisms have been associated with both decreased and increased agonist-induced desensitization of β2-AR-mediated cyclic adenosine monophosphate response [17], the changes in the receptor activation kinetics or downregulation of the receptor [18,19,20,21]. In our previous paper, we showed that polymorphism at position 16 and 27 may be important for the process of ligand association and dissociation to/from the receptor. The amino acid residues 16 and 27 lie at the *N*-terminus of β2-AR, which may be involved in the capture and temporary binding of the ligand molecules [22].

Another naturally occurring variant of β2-AR polymorphism, Thr164Ile, is correlated with a risk for diabetes and obesity as well as a differential therapeutic response to asthma and cardiovascular drugs [23]. In receptors containing the Ile164 variant, a significant decrease in basal and epinephrine stimulated adenylate cyclase activity was observed due to faulty coupling of the receptor to the stimulating G protein, Gs and impaired sequestration promoted by the agonist. The Ile164 variant also has a lower binding affinity for epinephrine compared to Thr164. Therefore, this amino acid change may be associated with a diminished response to the long-acting β2-agonist salmeterol. [23]. The in vivo studies showed that an increase in heart rate and contractibility mediated by β2-AR in response to terbutaline is blunted in individuals heterozygous for Ile164 compared with those homozygous for Thr164 [24]. Additionally, a potential association between the Ile164 variant and hypertension [25] and coronary artery disease [26] was found. This polymorphism is much rarer than that at positions 16 or 27 (40–50%), with an allelic frequency of about 4%, but is still interesting as the amino acid 164 is located in the fourth transmembrane-spanning domain of the receptor and is adjacent to conserved residues, serines 161 and 165, which are involved primarily in TM4 (4th transmembrane helix) packing and thereby maintain the stability of the receptor. Structural perturbations in this region of β2-AR directly affect receptor expression and agonist-dependent activity [27]. Moreover, in an inactive receptor Thr164 can create the hydrogen bonds with conserved serines 203 and 207 located on TM5. These residues are directly involved in agonist binding and receptor activation; thus, it can be assumed that Thr164 may affect the interaction with the ligand and activation of the receptor.

**Figure 1:**
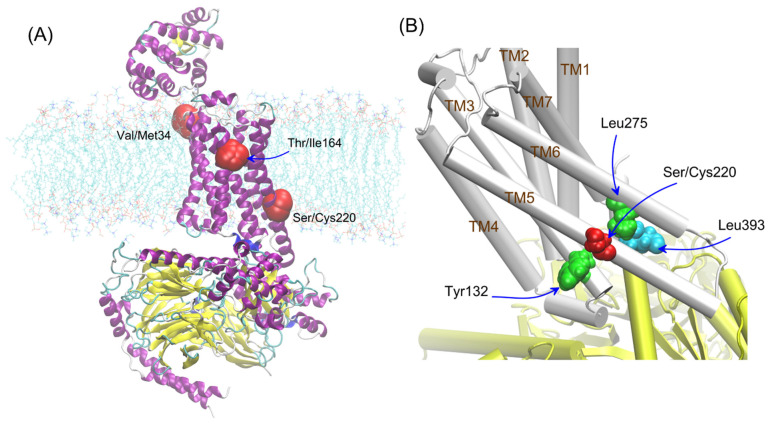
(**A**) The three studied polymorphisms and the corresponding amino-acid residues (in red) shown for the β2-AR molecule embedded in lipid bilayer and bound to Gs protein. The picture has an illustrative purpose and was prepared on the basis of the available crystal structure (PDB: 3SN6), used to prepare molecular models in our study. The color code refers to the secondary structure of protein: α-helices are highlighted in purple, β-sheets in yellow while coil regions in cyan. The polymorphic sites are represented by red spheres. (**B**) The schematic illustration of the crucial amino-acid residues interacting with Ser/Cys220 of β2-AR (in red), i.e., Tyr132 (TM3) and Leu275 (TM6) (in green) as well as Leu393 (Gs, in cyan). The molecules of β2-AR and Gs are shown in cartoon representations in white and yellow, respectively.

The other polymorphisms of β2-AR have not been studied so far, especially variants of the β2-AR polymorphism occurring in the transmembrane (Val34Met, Ser220Cys) parts of the receptor (see Figure 1A). The polymorphic variants of β2-AR differing by position 220 (Ser220Cys), located on TM5, i.e., the region which is subjected to conformational changes during the receptor activation and, at the same time, is relatively close to the attachment site of G protein (Figure 1B). The frequency of appearance the cysteine residue at the 220 position of β2-AR in human population is approx. 25/10,000 (according to the UniProtKB and NCBI (The National Center for Biotechnology Information) databases).

Another rare, nonsynonymous variant resulting in a valine-to-methionine amino acid change at position 34 (Val34Met) has a MAF (Minor Allele Frequency) about 1% [28].

As confirmed by a series of in vitro and clinical studies, individual variations in physiological responses, expression and function of β2-AR, as well as individual differences in response to drugs that act on these receptors, which may relate to the polymorphic variants of the receptor. However, the reasons for such differences and their interpretation at the scale of the molecular processes remain unclear. Thus, the main aim of the current work was to check whether any of the set of natural polymorphisms of the β2-AR associated with transmembrane part of the receptor (i.e., Val34Met, Thr164Ile and Ser220Cys) could significantly influence of the structure- or thermodynamics-mediated function. In particular, we considered whether the mutations corresponding to any of the above polymorphs affect the strength of β2-AR interactions with lipid bilayer and Gs protein as well as the local conformation of the receptor molecule. Moreover, we estimated the magnitude of free energy change associated with the β2-AR-Gs coupling.

In order to achieve these aims a series of molecular dynamics (MD) simulations were performed, either at all-atom (AA) or coarse-grained (CG) resolution. The regular, unbiased MD simulations were accompanied by enhanced-sampling protocols (nonequilibrium pulling simulations and thermodynamic integration).

The polymorphs were considered according to the environment in which their influence could be expected. All of them were located close to the vicinity of the lipid bilayer; thus, they were all investigated in the context of the lipid bilayer-β2-AR interactions. For adrenergic receptors, a potential dimer interface involving 1st transmembrane domain (TM1) was identified in the crystal structures of the β2-AR; thus, residue 34 (TM1) was taken into account in our studies [29]. The residue 164 is located near the binding cavity of β2-AR and it can affect the ligand binding [30]. Additionally, residue 220 is located close to the Gs/β2-AR interface created during the β2-AR-Gs coupling (see Figure 1). For this reason, this polymorphism seems to be potentially significant for β2-AR-mediated signaling. Thus, the involved interactions were considered by taking into account the two possible states of β2-AR, i.e., either unbound or in complex with Gs.

The simulations were concerned with a series of different β2-AR-containing systems, including: (i) Gs-free β2-AR molecule with three different models applied to represent the 3rd intracellular loop (ICL3) (simulations at the AA level); (ii) Gs-free β2-AR molecule with clipped model representing ICL3 (CG simulations); and (iii) Gs-bound β2-AR molecule with clipped model representing ICL3 (AA and CG simulations).

## 2. Results and Discussion

### 2.1. Structural Characteristics

First of all, it is worth noting that our simulated systems contained the receptor molecule without any ligand in its binding cavity. This is mainly because we intended to separate the two distinct aspects of polymorphism, i.e., (i) its influence on the intrinsic conformational properties of the receptor as well as interactions of the receptor with its environment (Gs protein and lipid bilayer) and (ii) possible effects on the ligand binding. The current article is focused on issues related to the former point. The results of our recent, preliminary studies indicate that ligand binding may be affected by some of the polymorphism types; however, this is heavily dependent on the considered ligand. Thus, we prefer to simplify our considerations by neglecting the presence of ligands in the binding cavity. This simplification is partially justified by: (i) the slow timescale associated with the activation–deactivation path; (ii) presence of active conformations of the receptor even in the absence of any ligand [31].

The results described in this subsection concern the receptor containing the ‘clipped’ model of ICL3. This concerns both AA and CG simulation results.

**Figure 2:**
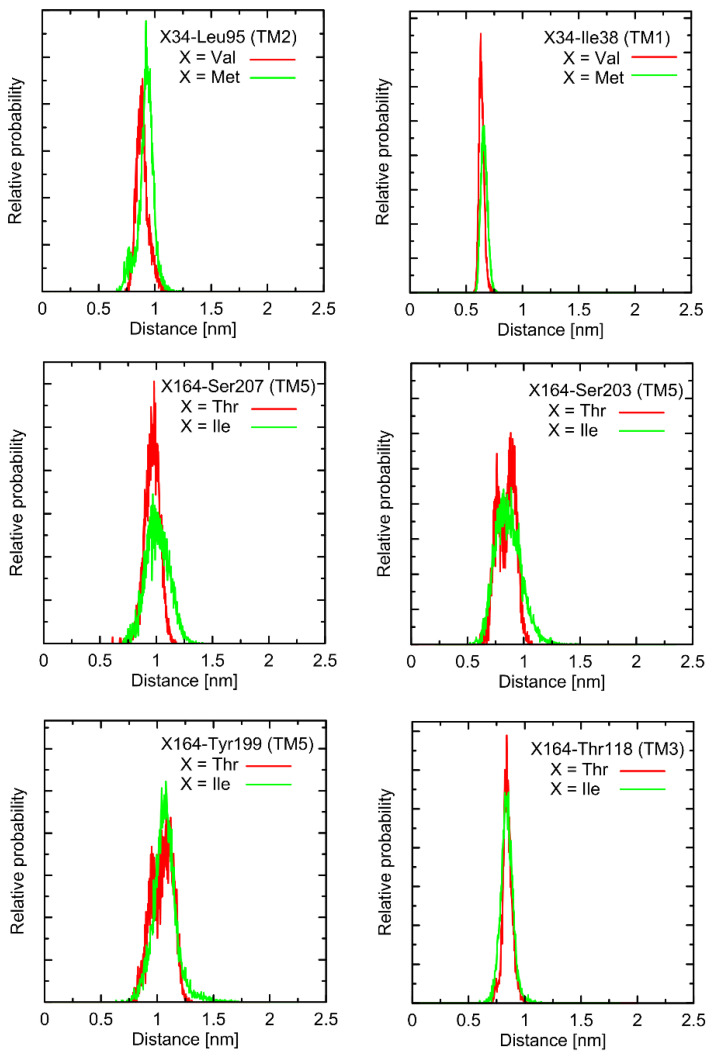
Radial distribution functions (RDFs) calculated from the unbiased molecular dynamics trajectories (100 ns run at the all-atom resolution, clipped model of Gs-free receptor) for the selected atom pairs of the system (β2-AR in unbound form). The distances were considered with respect to C_α_ atoms of either polymorphic site (i.e., 34 or 164) and the C_α_ atoms of selected, structurally-closest residues.

**Figure 3:**
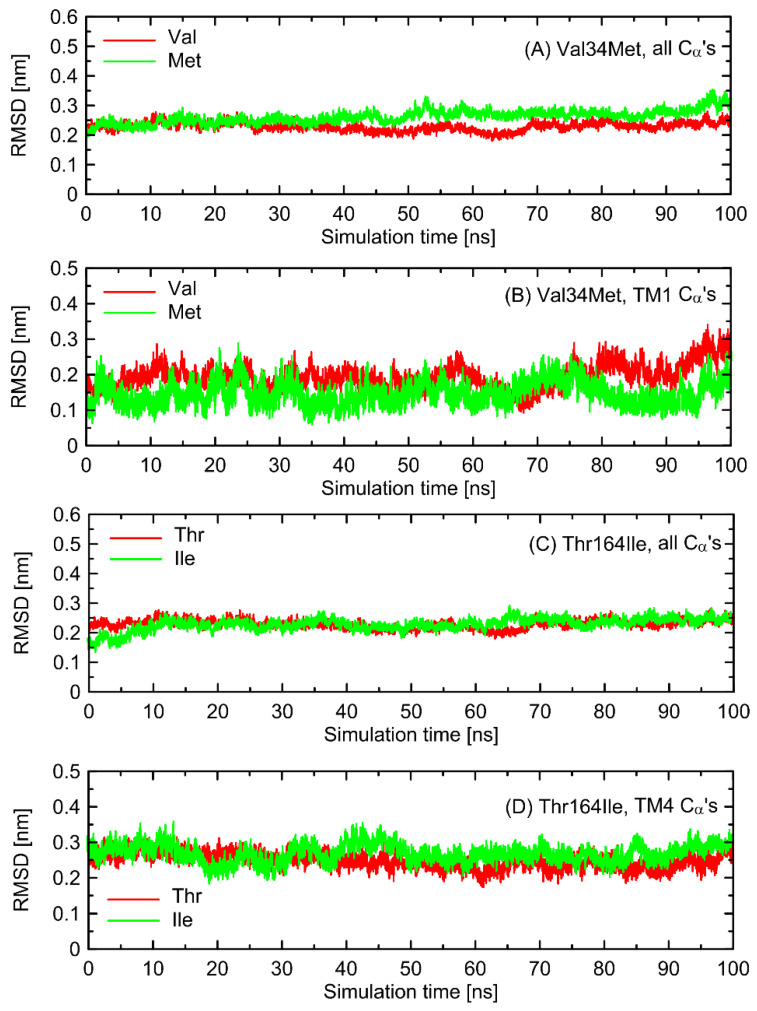
The values of the root-mean-square deviation (RMSD) parameter calculated for the polymorphs differing by residue type at positions 34 and 164. The reference structure was always Gs-bound β2-AR (PDB: 3SN6). The data concern the all-atom molecular dynamics simulations of Gs-free β2-AR (100 ns). (**A**,**C**) RMSD calculated on all C_α_ carbon atoms in the β2-AR structure. (**B**,**D**) RMSD calculated on C_α_ carbon atoms belonging to the transmembrane helix where the given polymorph appears (i.e., 1st or 4th transmembrane helix, TM1 and TM4, respectively).

In the first stage of the study, the possible alterations in the conformational properties of the receptor induced by natural polymorphs at positions 34 and 164 were studied. We have found that no systematic, large-scale changes occurred either in the conformation of the whole receptor molecule or even at more local scale. This statement relies on both the all-atom and coarse-grained simulations and is based on the comparison of the RMSD (root-mean-square deviation) parameter values as well as on the several structural descriptors capable of illustrating the receptor activation process (e.g., radial distribution functions, RDFs, calculated for pairs of C_α_ atoms in the vicinity of positions 34 and 164). The exemplary data from AA simulations are shown in Figure 2 and Figure 3. The RMSD parameter represents the deviation of a given structure in a reference to another, fixed structure; the larger values of RMSD denote larger deviations from a reference structure, whereas 0 value represents an ideal match between both structures. RDF is a measure of the probability of finding an atom at a certain distance away from a given reference atom.

As shown in Figure 2 and Figure 3, mutations at either position 34 or 164 do not seriously influence the interatomic distances between the polymorphic site and the nearest residues, including both those belonging to the same helix as the polymorphic site and to the neighboring ones. The mentioned distances concern the protein backbone; however, some sidechain-related rearrangements may occur, which is a direct consequence of different molecular features of involved amino-acids. In spite of that, such rearrangements do not alter the structure of either the whole receptor or even the helix at which the polymorphic site is located, as indicated by the RMSD values (Figure 3).

Note that these findings indicate a minor influence of polymorphisms at positions 34 and 164 on the conformational features of the receptor, if relying only on the study of ligand-free β2-AR. The potential ligand-mediated alterations are not possible to exclude and, while not studied in the present work, are postponed to our future investigations.

**Figure 4:**
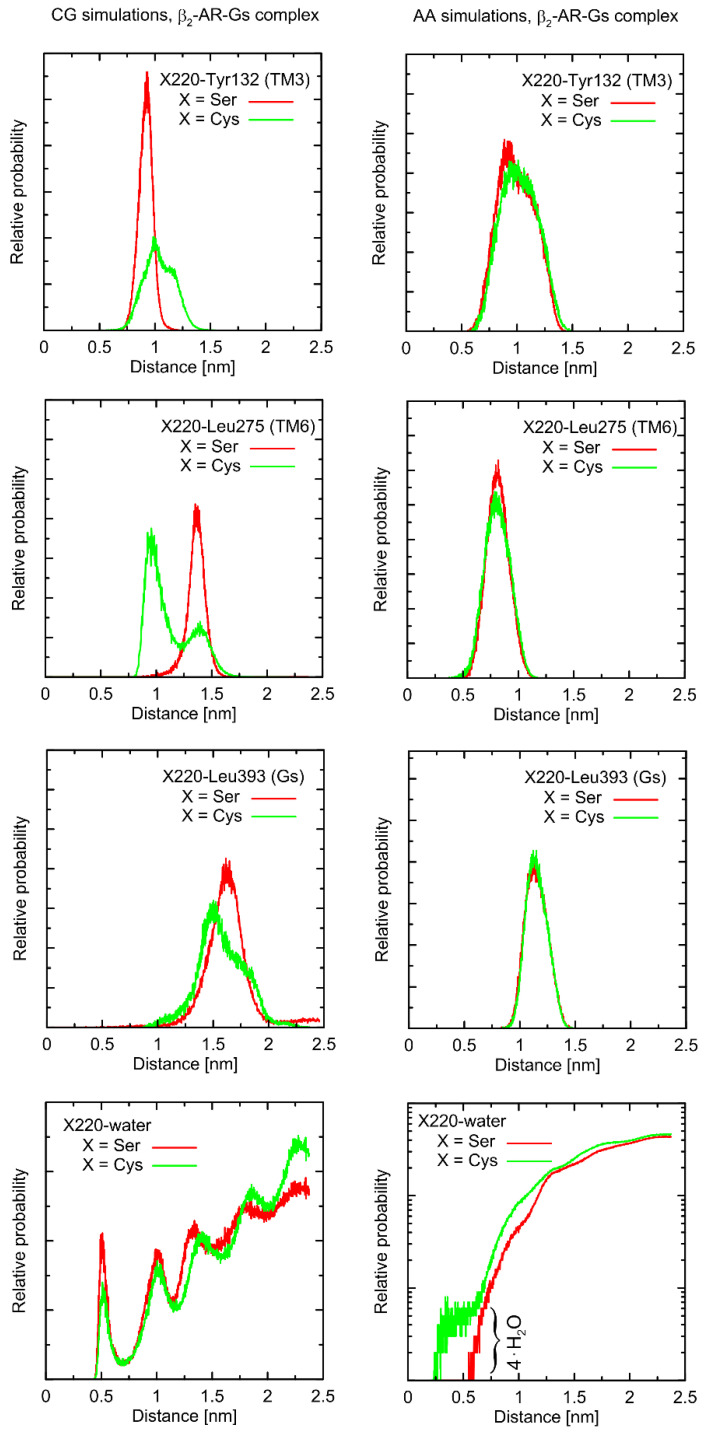
The radial distribution functions calculated from the MD trajectories (either at the all-atom, AA, or coarse-grained, CG, resolution) for the selected elements of the system (β2-AR in complex with Gs protein). The distances were considered with respect to sidechains of amino-acid residues (center-of-mass approach) or water molecule/bead. For clarity, the logarithmic scale was applied in one of the lowest panels. Simulations lasted 100 ns (AA) or 500 ns (CG) and the clipped model of Gs-bound receptor was used.

**Figure 5:**
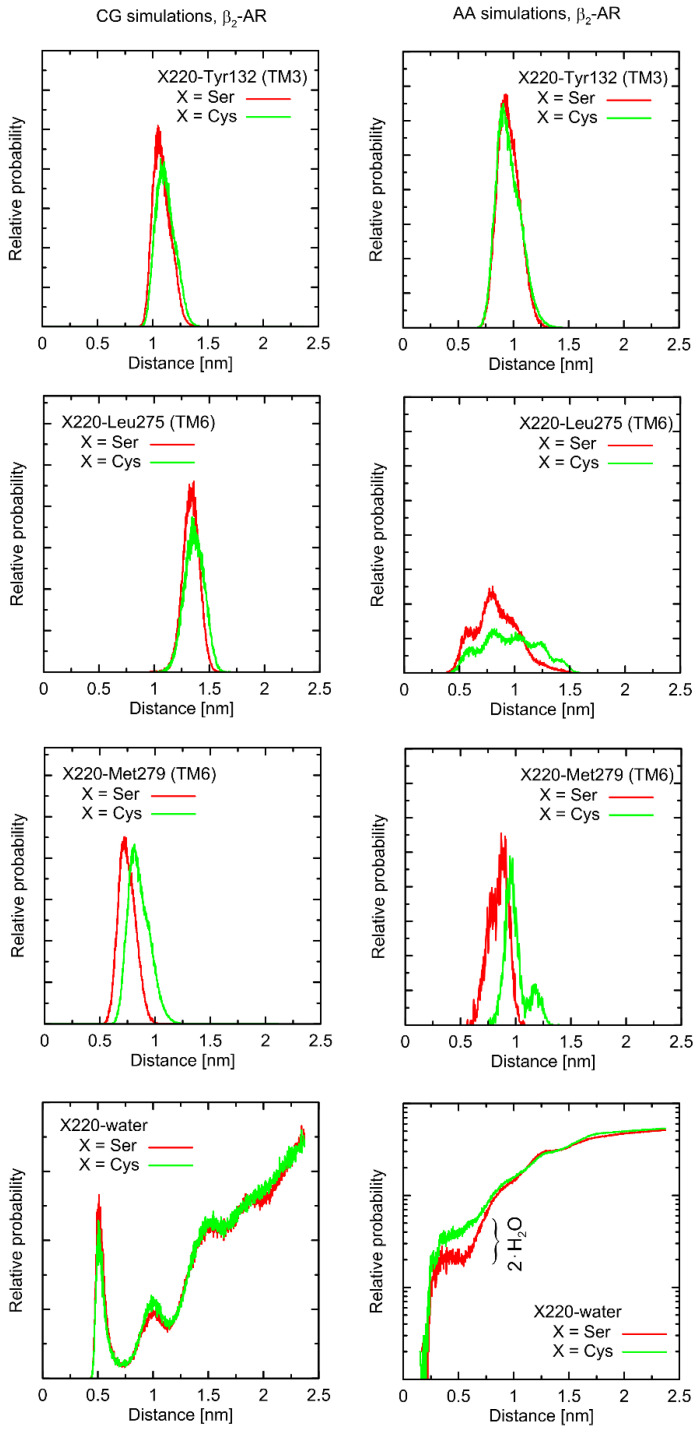
The radial distribution functions calculated from the MD trajectories (either at the all-atom or coarse-grained resolution) for the selected elements of the system (unbound β2-AR). The rest of details as in Figure 4.

Considering the Ser220Cys polymorphism, there exists a series of conformational alterations induced by the presence of the Gs protein (Figure 4) when compared to the unbound β2-AR molecule (Figure 5). Even more importantly, the calculations of RMSD carried out for unbound forms of the receptor with respect not to the initial structures, but to the same, equilibrated, β2-AR structure, present in the β2-AR-Gs complex, revealed a series of differences which seem to be correlated with the type of polymorph at position 220 (this is discussed later).

The example of the former observation is the stabilizing effect of the attached Gs molecule on the Ser/Cys220-Leu275 distance (as shown Figure 4). This results from a disturbed conformational landscape of β2-AR and from creating a network of strong intermolecular interactions at the β2-AR/Gs interface. Alterations of this type are expected and will not be discussed in detail, as they are not directly connected to the polymorphism effect.

From a more local perspective, we observed only few notable and polymorphism-related variations in conformational properties of the local environment of mutation points. In most cases, such potential changes are not likely to play any signaling-related role. For instance, this is the case of the Val34Met and Thr164Ile polymorphs: due to their distant location from the G protein binding site or the lack of involvement in the ligand binding. However, the Ser220Cys polymorphism is located close to the β2-AR/Gs interface in the β2-AR-Gs complex; thus, this particular case is discussed in more detail.

First, let us note that predictions offered by either atomistic and coarse-grained simulations are very similar, and the differences can usually be fully explained by different definitions of the center of interactions inherent to each of theoretical approaches. This includes, e.g., the two conformational states predicted by the CG approach in the case of Leu275 and Cys220 and the β2-AR-Gs complex, in contrast to atomistic simulations, where only one state is predicted. This difference may partially be explained by the larger dimensions of the Cys CG bead, which is capable of creating a direct contact with the Leu bead. In the case of atomistic resolution, the possible interactions are more complex and the smaller water molecules prevent the formation of such a contact. The most systematic difference, considering the consistency of the CG and atomistic simulation results, is connected with the solvation state of the sidechain of residue 220. More precisely: the CG results predict the opposite trends in solvation degree in comparison to the atomistic ones. This can also be explained by the diverse definitions of water molecule, which, in the case of CG parameters, is only a part of one water ‘bead’, representing four water molecules. 

The Ser220Cys polymorphism, according to CG simulation, is capable of slightly altering the distances between TM5 (i.e., transmembrane domain on which residue 220 is located) and TM3 (β2-AR^Cys220^ polymorphism induces the increase of both this distance and its fluctuations) as well as between TM5 and TM6 (decreasing the distance correlated with appearance of the distinct conformational state). However, both these changes concern only the β2-AR-Gs complex and do not appear in the unbound form of β2-AR (Figure 4 and Figure 5). Note that the Ser-containing polymorph exhibits the values of both above-mentioned descriptors (i.e., TM5-TM3 and TM5-TM6 distances) closer to those corresponding to Gs-bound form of β2-AR. This may suggest that the presence of Cys disfavors the binding of Gs for structural reasons. As the above-reported changes are not directly reflected by the atomistic simulation results, they should be interpreted with caution.

Atomistic simulations predict nearly none of the polymorphism-related differences in the considered interatomic distances for the β2-AR-Gs complex and the closest vicinity of the residue at position 220. On the other hand, several corresponding differences are observed for the unbound form of β2-AR. They are discussed later in detail but let us now briefly point out that those (minor) differences (e.g., the small shift of the average value and the fluctuation magnitude in the case of the TM5-TM6 distance) suggest that the β2-AR^Ser220^ polymorph structure is closer to that inherent to the Gs-coupled β2-AR. This again may be interpreted as a small structural contribution favoring the Gs binding by the β2-AR^Ser220^ polymorph. This observation is qualitatively (but not quantitatively) in line with the results based on the CG simulations.

Finally, atomistic simulations predict a systematic alteration in the solvation of the sidechain at position 220; Cys exhibits a larger solvation shell, encompassing either four or two water molecules, in comparison to Ser, in the case of the β2-AR-Gs complex or unbound β2-AR, respectively. This difference has its source in both the different dimensions and chemical characters of the considered sidechains and their diverse conformational behavior (see discussion below).

### 2.2. Conformational Switches

The main part of results described in this subsection concern the receptor containing the ‘clipped’ model of ICL3 (data illustrated in Figure 6, Figure 7 and Figure 8 and Figures S1–S3). The results obtained for alternative loop models (open loop and fully modeled ICL3) are illustrated in the Supplementary Materials (Figures S4–S7). The results for β2-AR-Gs complex with the use of clipped model are shown in Supplementary Materials (Figures S8 and S9).

**Figure 6:**
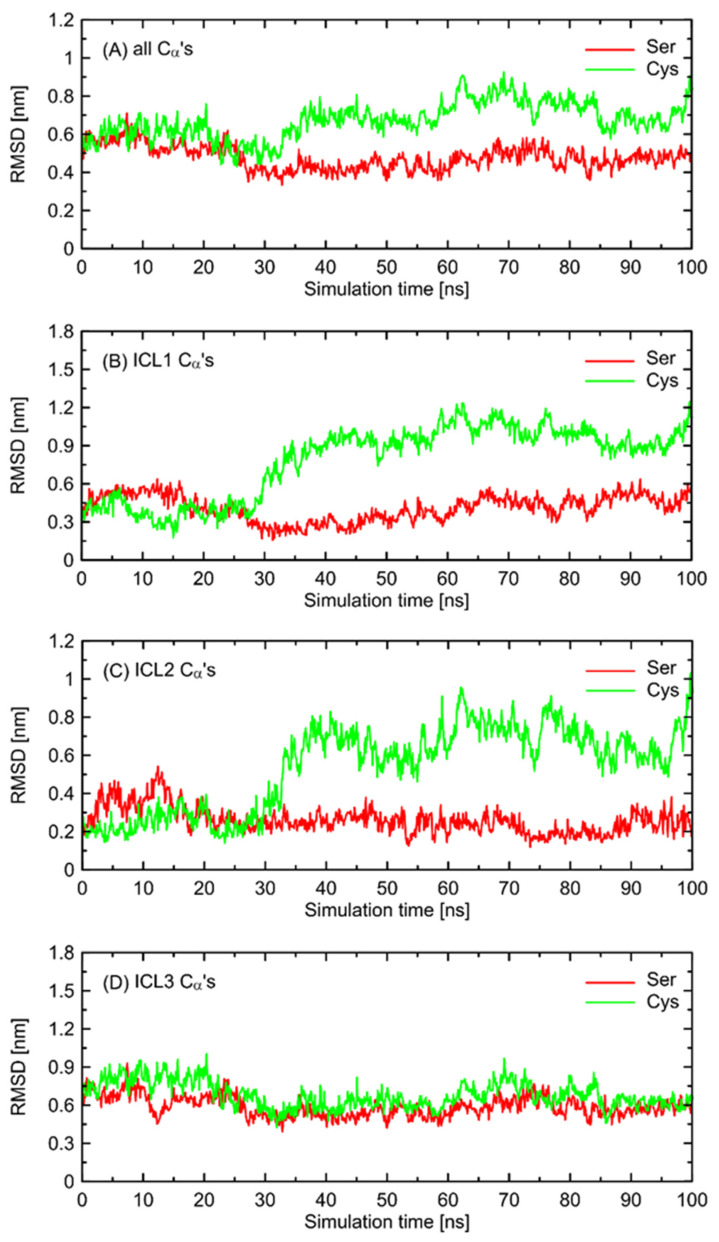
The values of the root-mean-square deviation (RMSD) parameter calculated for β2-AR^Ser220^ and β2-AR^Cys220^ polymorphs with respect to the structure of Gs-bound β2-AR. The data concern the all-atom molecular dynamics simulations of Gs-free β2-AR (100 ns, clipped model of the 3rd intracellular loop, ICL3). (**A**) RMSD calculated on all the C_α_ carbon atoms present in all three ICLs. (**B**–**D**) RMSD calculated on the C_α_ carbon atoms belonging to the following fragments of the receptor structure: (**B**) ICL1 (Lys60-Thr66); (**C**) ICL2 (Phe133-Asn148); (**D**) ICL3 (Ser/Cys220-Glu268).

In order to further investigate the monitored, structural differences that may favor the Gs-bound-like conformation, we checked the root-mean-square deviation (RMSD) values for both Ser- and Cys-containing polymorphs of the Gs-free form of β2-AR with a reference to the Gs-bound structure. The results are given in Figure 6 and show a significant conformational alteration concerning β2-AR^Cys220^ polymorph. Such alteration is correlated with the deviation of the whole structure further away from the Gs-compatible conformation of β2-AR. Thus, it is again confirmed that β2-AR^Cys220^ polymorphs may be structurally less similar to the Gs-bound form and, thus, less prone to Gs binding. Close inspection of the particular fragments of the receptor that may contribute to the observed deviation in the RMSD values revealed that they are located in the intracellular part of the receptor, being an interface capable of binding the Gs protein. More precisely, the conformational changes occur within the 1st and 2nd intracellular loops (ICL1 and ICL2, defined as in caption to Figure 6). Interestingly, no analogous structural alterations expressed by the RMSD values are connected with ICL3, which is the closest to the polymorphic site.

**Figure 7:**
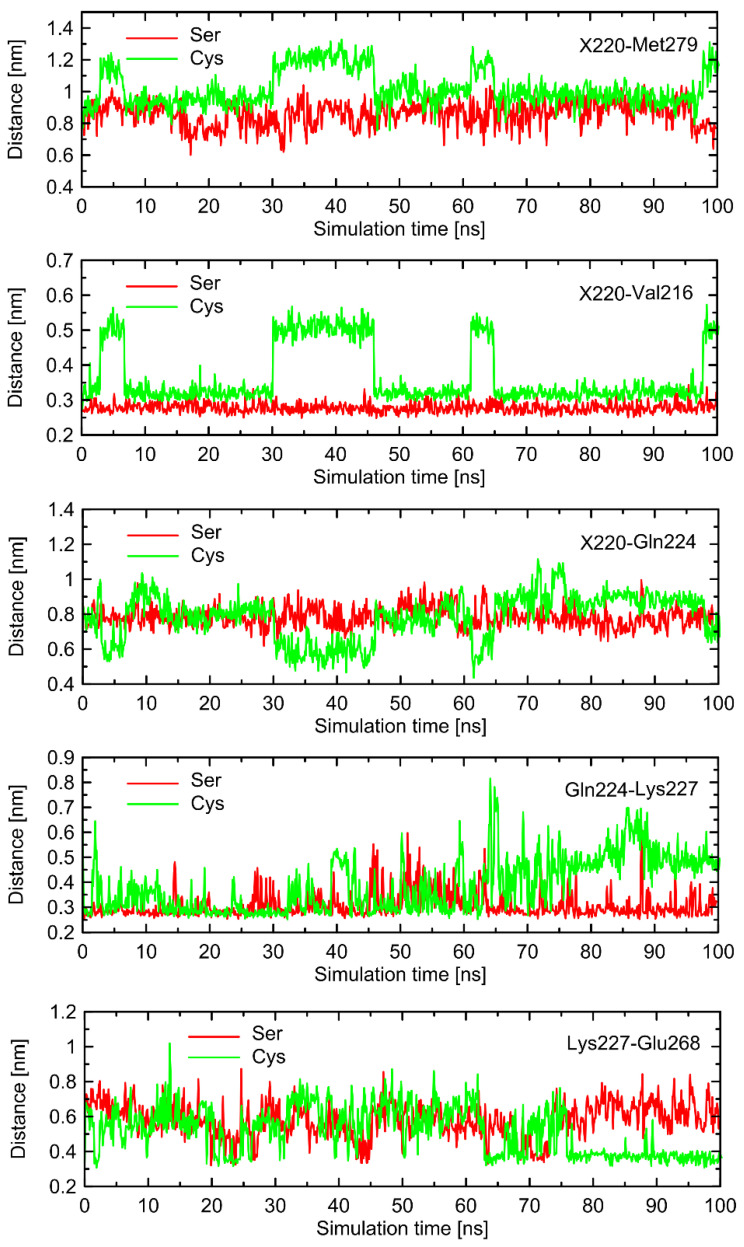
The selected, time-dependent interatomic distances identified in the analysis of molecular dynamics (MD) simulations as potentially important for polymorphism-related effects in G-protein binding by β2-AR. The data were obtained from the 100 ns-long, unbiased all-atom MD simulations of the Gs-free β2-AR with the clipped model of the 3rd intracellular loop (ICL3). The shown distances correspond to structural rearrangements occurring within ICL3. X = Ser or Cys.

**Figure 8:**
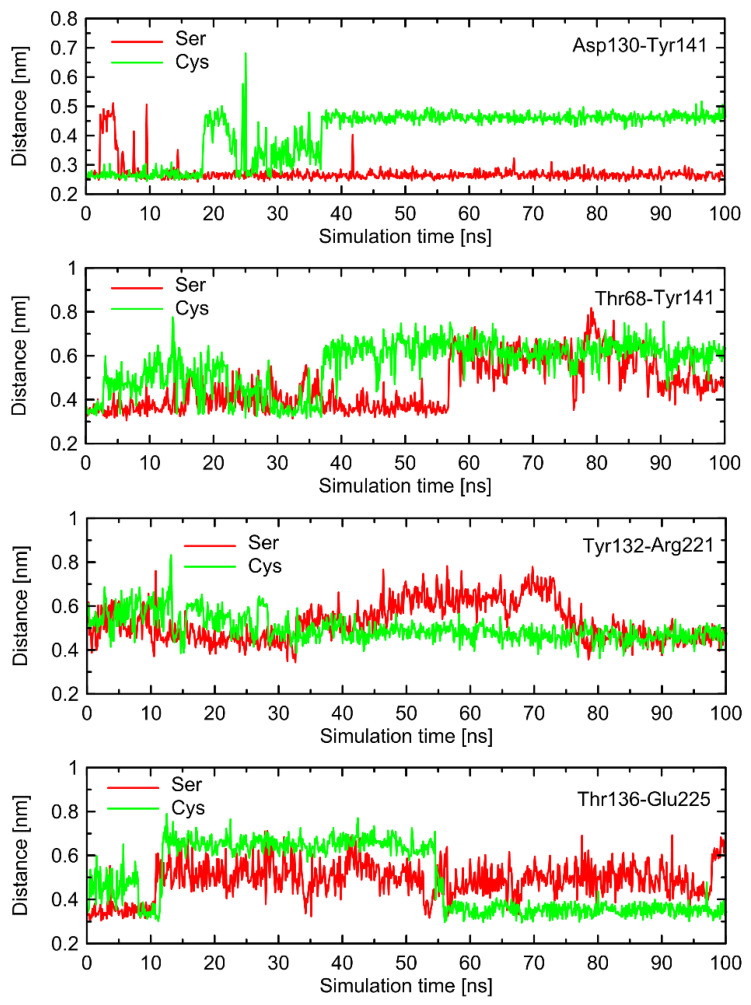
The selected, time-dependent interatomic distances identified in the analysis of molecular dynamics (MD) simulations as potentially important for polymorphism-related effects in G-protein binding by β2-AR. The data were obtained from the unbiased all-atom MD simulations of the Gs-free β2-AR (100 ns run, clipped model of the 3rd intracellular loop, ICL3). The shown distances correspond to structural rearrangements occurring within ICL1 and ICL2.

In order to find the source of the polymorphism-related structural altercations in the unbound form of β2-AR, which manifest themselves in RMSD values, we identified several interatomic distances that do not necessarily involve the polymorphic site, but seem to undergo the largest polymorphism-dependent variations. The results are given in Figure 7 and Figure 8. The structural changes in ICL3 correlating to the transformation of Ser220 into Cys220 were not reflected by any abrupt variations of the RMSD values calculated for C_α_ atoms within this loop (Figure 6). The probable reason for that is that such changes lead to the set of structures which are equally distant from the reference structure either before or after conformational transitions. However, conformational rearrangements in the vicinity of ICL3 are still notable and involve: (i) possible reorientations of the Cys220 sidechain, which can interact with either the backbone fragment of Val216 or the sidechain of Gln224—the latter scenario is connected with an increased distance to TM6 (in particular, to Leu275 and Met279), as mentioned previously; (ii) the conformationally-rearranged Gln224 sidechain, interacting with Cys220 is unable to form stable contacts with Lys227, and the corresponding distance increases; and (iii) Lys227, not engaged in contact with Gln224 is capable of creating the interactions with Glu268, ultimately forming an ionic bridge.

Steps (i)–(iii) define the conformational rearrangements that constitute the polymorphism-dependent molecular switch acting only in the case of β2-AR^Cys220^. The stable orientation of the Ser220 sidechain does not allow the completion of the remaining steps of the analogous, hypothetical conformational transition in β2-AR^Ser220^.

In order to check whether the above-described findings are reproducible across a set of independent MD simulations, we carried out two additional series of simulations, initiated from different initial velocities. The results are illustrated in Figures S1–S3 (Supplementary Materials). The analysis of repeated runs indicated that, qualitatively, the same results were obtained. The quantitative results appear to differ from run to run; however, we did not perform any quantitative analysis of the observed switches, thus, most essential conclusions remain unchanged. The observed similarities between independent runs include: (i) larger deviations of the RMSD values were calculated for ICLs in reference to the Gs-bound receptor for β2-AR^Cys220^ in comparison to β2-AR^Ser220^, contributed mainly by ICL1 and ICL2; (ii) analogous behavior of molecular switches initiated from the conformational movements of Cys220, but not Ser220, correlated with some rearrangements within ICLs. Interestingly, in the case of one of the runs, the Cys220 sidechain displayed smaller flexibility, which was correlated with smaller alterations of the RMSD values and other parts of molecular switch (e.g., the Lys227-Glu268 ionic bridge). This is in line with statements that the observed deviations in the structure of ICLs are associated with the Cys220 conformation.

Interestingly, a similar type of conformational behavior is observed in the case of β2-AR^Cys220^ coupled with Gs protein. There, the series of molecular switches located on the TM5 acts in an analogous manner in comparison to the Gs-free β2-AR. However, in this case, the correlation with the behavior of switches not directly involved in interactions with Cys220 is not so evident. This is because the stabilizing effect of the bound Gs protein which restricts the conformational movements within ICLs. As a consequence of this fact, some parts of the conformational rearrangements are not sensitive to the polymorph type (e.g., the Lys227-Glu268 ionic bridge) and some (especially those within ICL1 and ICL2) exhibit distinct behavior (e.g., Asp130-Tyr141). Therefore, it can be concluded that the Ser/Cys220-characteristic behavior is present in both Gs-bound and Gs-free receptors, but its propagation into the further parts of intracellular part of the receptor is dependent on the presence or absence of the attached Gs protein. The corresponding data are illustrated in Figures S8 and S9.

The above-discussed results rely on the receptor model containing ‘clipped’ ICL3. Although the polymorphic site and the remaining residues which create the rest of molecular switch are not a part of missing fragments in the crystal structure of β2-AR, they are close enough to consider the potential influence of the approach being applied to treat missing ICL3 in the obtained results. Therefore, the analysis of the conformational properties of the same series of residues (in analogy to Figure 7 and Figure 8) has been performed for two other models of ICL3. More precisely, the ‘open loop’ and ‘fully-modelled loop’ approaches were applied (see Section 3 for details) and the results are illustrated in Figures S4–S7 (Supplementary Materials).

The results concerning the influence of the Ser220Cys polymorphism seem to be fairly independent on the chosen loop model. In general, the results obtained for different models of loop are reproducible in a qualitative, but not quantitative, manner. This type of agreement is acceptable due to the fact that our analysis of molecular switches associated with the Ser220Cys polymorphism is also of a qualitative nature. Regarding the differences between the results originating from systems with different loop models, it is also worth noting that the deviation between sets of results is of similar magnitude in comparison to the results obtained for the original system in triplicated MD runs. To summarize, independently on the loop model, we observed: (i) the conformational rearrangements within the Cys220 + Val216 + Gln224 part of the molecular switch; (ii) the increased tendency to keep the Lys227-Glu268 ionic bridge closed, observed in the case of Cys220 in comparison to Ser220; (iii) qualitative agreement between tendencies in the values of selected interatomic distances located either within the above-mentioned residues or within ILC1 and ICL3; (iv) increased deformation of the intracellular structure for β2-AR^Cys220^ in comparison to β2-AR^Ser220^ when the Gs-bound receptor is taken as a reference, contributed mainly by atoms belonging to ICL1 and ICL3.

**Figure 9:**
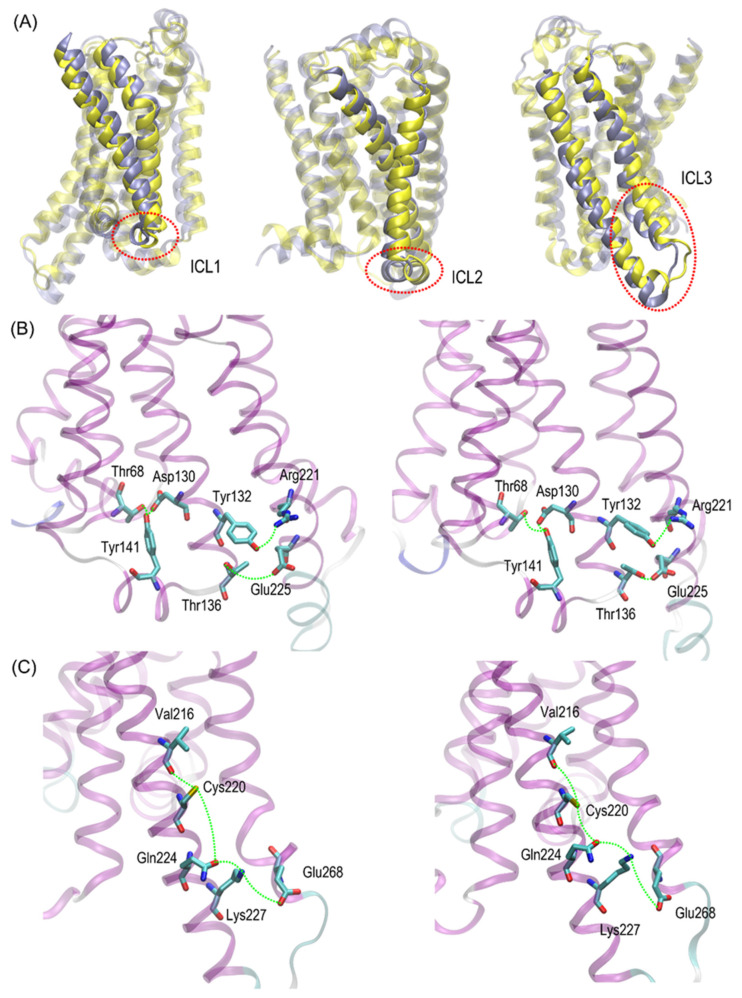
(**A**). The graphical illustration of the structural, polymorphism-dependent alteration observed during all-atom molecular dynamics simulations of either Ser220- or Cys220-containing β2-AR. The superposed structures of β2-AR^Ser220^ and β2-AR^Cys220^ are shown in light violet and in yellow, respectively. The regions marked by red, dotted lines correspond to intracellular loops ICL1, ICL2 and ICL3. (**B**,**C**). The graphical illustration (green dotted lines) of the most essential, interresidual contacts undergoing the largest polymorphism-related variations (see Figure 7 and Figure 8) within either ICL1 and ICL2 (**B**) or ICL3 (**C**).

Figure 9 illustrates the limiting conformations of the selected regions of the β2-AR^Cys220^ polymorph of β2-AR, identified in the MD simulations and reflecting the polymorphism-related structural alterations mentioned before. The β2-AR^Ser220^ polymorph was not shown there; however, its dynamic structure is always closer to the conformation shown on the left-hand-side panels of Figure 9 due to a more stable conformation of the Ser220 sidechain.

The conformational changes occurring within ICL1 and ICL2 for β2-AR^Cys220^ have a much larger influence on the RMSD values (Figure 6) and act systematically toward larger deviations from the Gs-compatible structure of β2-AR. Those changes have been identified as the results of disrupted interactions within both ICLs as well as between ICL2 and ICL1. In the former case, the crucial, hydrogen bonding-mediated contact between sidechains of Tyr141 and Asp130 is disrupted and the corresponding interatomic distance increases. An analogous situation occurs in the case of Tyr141 and Thr68 (located on TM2), which also is correlated with the increase in the Tyr141-Thr68 distance. Both those rearrangements lead to the movement of ICL2 further away from the intracellular entrance to the receptor channel. The reoriented loop comes closer to one of the edges of ICL3 and lower part of TM3. This enables the creation of a series of closer contacts, e.g., the one between Thr136 and Glu225. Several further corresponding distances between ICL2 and ICL3 are stabilized (e.g., Tyr132-Arg221) in comparison to β2-AR^Ser220^. Finally, the lost Tyr141-Thr68 contact is responsible for the rearrangement of ICL2, in comparison to both the Gs-compatible structure and β2-AR^Ser220^.

The dynamic nature of the reorientation of the Cys220 sidechain hinders the unequivocal identification of direct causes and consequences of all observed conformational rearrangements. Nevertheless, the correlation between structural alterations and polymorphism is clear and allows the conclusion that the Ser220Cys polymorphism is capable of changing the free energy landscape, describing the intracellular conformation of β2-AR to a non-negligible extent.

In summary, we have identified several conformational alterations dependent on the Ser220Cys polymorphism and are located within the intracellular part of the receptor. From the perspective of Gs protein binding, the structure of β2-AR^Ser220^ resembles the Gs-bound conformation of the receptor to higher extent.

### 2.3. Energetic Characteristics

The energetic characteristics relies on the calculated values of free energy, accompanied by the X → Y transformation, where X and Y are the corresponding pairs of amino-acid residue. As the single value of such free energy change is nearly meaningless in terms of physical processes, an additional reference value is required to fully describe the limiting states of a given process. Here, we decided to consider the free energies that can be attributed to: (i) the process of the transfer of the receptor from the aqueous solution to lipid bilayer (immersion); and (ii) the binding of the Gs protein to β2-AR, according to the pattern observed in the experimental, XRD data.

The obtained free energy values (Table 1) have a rather minor magnitude, not exceeding 5 kJ/mol, which clearly suggests a small influence of all types of mutation for the considered process. Notably, the absolute free energy changes are usually of order of simulation errors. Interestingly, the results corresponding to the interactions with lipid bilayer do not exhibit systematic signs and are dependent on the composition of lipid bilayer. Although a detailed analysis exceeds the scope of the present paper, this finding indicates the importance of the lipid bilayer composition on the reproducibility of the results aimed at assessing the accurate influence of point-mutation on the lipid bilayer-related interactions.

The free energy changes collected in Table 1 have a relative character, i.e., the description of the differences in free energy changes depend upon a given mutation. However, without any knowledge of the reference energy, it is hard to state whether the calculated (apparently minor) difference is of any relevance to the considered process. This is especially true with respect to the processes associated with the β2-AR-Gs coupling, being paramount to β2-AR-mediated signaling.

**Figure 10:**
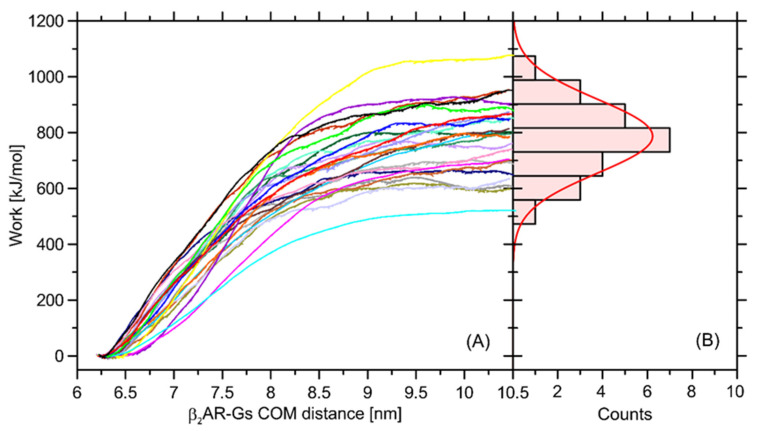
(**A**) The profiles of work recovered from 24 independent nonequilibrium pulling simulations aimed at the estimation of the β2-AR-Gs binding free energy by using the Jarzynski relation. (**B**) The histogrammed work values calculated in the previous step exhibit a Gaussian-like distribution with average works equal to 787 kJ/mol and standard deviation equal to 132 kJ/mol. The data were generated by using all-atom molecular dynamics simulations.

In order to at least approximately determine the magnitude of influence of the Ser220Cys mutation on the total β2-AR-Gs binding free energy, we performed a series of additional calculations relying on the nonequilibrium puling simulations (see Section 3 for details). The recovered distribution of the work values determined in a series of separate simulations display a Gaussian-like distribution, which is an indicator of sufficient sampling. The average free energy is equal to ~787 kJ/mol and the deviation corresponding to the Gaussian distribution is equal to 132 kJ/mol (Figure 10).

The magnitude of determined free energy is relatively high, which corresponds to the large area of contact between the coupled β2-AR and Gs molecules, but also may be a result of approximate representation of the dissociation process, which, in reality, may occur through a series of intermediate states and involve intra-Gs conformational rearrangements. The value of 787 kJ/mol largely exceeds our previous results, reported in ref. [32] and based on the coarse-grained molecular dynamics simulations. Furthermore, the binding free energy of an order of hundreds of kJ/mol largely over-exceeds the relative energy changes calculated with respect to the Ser → Cys transformation and collected in Table 1. Therefore, it can be concluded that Ser220Cys polymorphism does not significantly affect energetics of the β2-AR-Gs coupling.

## 3. Materials and Methods

### 3.1. All-Atom Simulations

The structures of either β2-AR or the β2-AR-Gs complex were based on the XRD structural data deposited in the PDB:3SN6 entry. The nanobody, lysozyme-fusion protein and co-crystallized agonist were removed from the structure. In those cases where a Gs-free receptor was considered, the Gs complex was removed as well. The three distinct approaches were used to treat the ICL3 structure (missing in the crystallographic data): (i) the ‘clipped’ model, where TM5 and TM6 terminal chain fragments are covalently attached to each other; (ii) ‘open loop’ model in which the loose ends of TM5 and TM6 are not covalently bound but treated as additional *N*- and *C*-termini; and (iii) the ‘loop’ model, where ICL3 is modeled by the MoMA-LoopSampler online server (moma.laas.fr, [33]) and its structure is explicitly present in the simulated system. The Gs-bound structure of β2-AR was always represented by the ‘clipped’ model of ICL3. The β2-AR molecule was placed in a rectangular simulation box of dimensions 12 × 12 × 14 nm^3^, immersed in the DPPC lipid bilayer and surrounded by the explicit water molecules and appropriate number of Na^+^ and Cl^−^ ions, neutralizing the charge whenever needed and elevating the ionic strength to 0.15 M. In the case of the β2-AR-Gs complex, the box size was increased to 15 × 15 × 18 nm^3^, whereas for the pulling simulations, the Z dimension of the box was additionally increased to 29 nm. The systems were prepared to multi-step geometry optimization and equilibration protocol, relying on the gradual unconstraining of the protein structure in parallel to applying the pressure control. The following steps were applied: (i) frozen protein, positional restraints with force constants equal to 1000 kJ/mol/nm^2^ on heavy atoms of lipids, 1 ns NVT simulation; (ii) positional restraints with force constants equal to 1000 kJ/mol/nm^2^ on each heavy atom of protein, 5 ns NPT simulation; (iii) positional restraints with force constants equal to 100 kJ/mol/nm^2^ on the protein backbone, 5 ns NPT simulation; (iv) positional restraints with force constants equal to 10 kJ/mol/nm^2^ on the protein backbone, 5 ns NPT simulation; and (v) unconstrained NPT simulation lasting 15 ns. The total duration of all equilibration stages was equal to 31 ns for all systems. After equilibration, the systems were subjected to the standard, unbiased MD simulations or to the free energy calculations (see details below).

All MD simulations were carried out with the GROMACS 2016.4 package [34] with within the all-atom CHARMM36 force field [35]. Periodic boundary conditions and the isothermal–isobaric ensemble were applied. The temperature was maintained close to its reference value (310 K) by applying the V-rescale thermostat [36], whereas for the constant pressure (1 bar, isotropic coordinate scaling) the Parrinello-Rahman barostat [37] was used with a relaxation time of 0.4 ps. The equations of motion were integrated with a time step of 2 fs using the leap-frog scheme [38]. The TIP3P model of water [39] was applied. The hydrogen-containing solute bond lengths were constrained by application of the LINCS procedure with a relative geometric tolerance of 10^−4^ [40]. The electrostatic interactions were modeled by using the particle-mesh Ewald method [41] with cut-off set to 1.2 nm, while van der Waals interactions (LJ potentials) were switched off between 1.0 and 1.2 nm. The translational center-of-mass motion was removed during every timestep separately for the solute and the solvent. The full rigidity of the water molecules was enforced by the application of the SETTLE procedure [42]. Production simulations were carried out for a duration of 100 ns and the data were collected every 2 ps.

The analysis of RMSD (root-mean-square deviation) was performed by using the *gmx rms* tool (part of GROMACS) and was concerned with the C_α_ atoms of selected parts of the β2-AR structure. The interatomic distances and RDFs (radial distribution functions) were calculated by using the *gmx rdf* tool, either for the selected pairs of the C_α_ atoms or groups of atoms belonging to sidechains. More precisely, the following atoms/groups of atoms were considered in the case of sidechains: S atom (Cys), O atom (Ser, Thr, Tyr, Gln), methyl group (Met), carboxyl group (Glu, Asp), amine group (Lys) and C_α_ atom (Arg). If more than one atom was considered, the center-of-mass of a given group was considered as a reference. The above description is also valid for the CG simulations and the role of atoms or groups of atoms is played by single CG beads.

### 3.2. Coarse-Grained Simulations

The initial protein structures for coarse-grained simulations were taken from the XRD structural data deposited in the PDB:3SN6 entry and converted to coarse-grained (CG) resolution by using the martinize.py script. As in the case of AA simulations, only the Gs complex and β2-AR were considered and the only treatment of the missing ICL3 fragment was applying the ‘clipped’ model. The nonpolarizable Martini model version 2.2 was employed [43,44] in the simulations. In all CG simulations, the elastic network [45] with default options, i.e., a force constant of 500 kJ mol^−1^ nm^−2^ and an upper cut-off of 0.9 nm, was employed for the transmembrane domains.

The β2-AR molecule and the β2-AR-Gs complex were placed in rectangular simulation box of dimensions 12.5 × 12.5 × 14.5 nm^3^ and 16 × 16 × 19 nm^3^, respectively. The immersion of β2-AR into lipid bilayer and the subsequent solvation in the MARTINI water was carried out by the insane.py script. The following compositions of lipid bilayer were considered: (i) DPPC; (ii) POPC; (iii) POPE; and (iv) model mammalian bilayer composed of the following compounds: POPC (molar fraction: 18%), POPE (21%), POPS (11%), cholesterol (34%) and palmitoylsphingomyelin (16%).

All CG MD simulations were carried out with the GROMACS 2016.4 package [34]. The reaction field electrostatics and Lennard-Jones potentials were shifted to zero at the cut-off distance of 1.1 nm. A dielectric constant of 15 was employed up to the cut-off length, after which it was given a value of infinity. The Verlet cut-off scheme was employed, as implemented in GROMACS. Temperatures of the protein(s), the lipids and the solvent were separately kept constant at 310 K with the v-rescale thermostat [36], while pressure was maintained semi-isotropically at 1 bar using the Parrinello–Rahman barostat [37] with a time constant of 20 ps and a compressibility of 3 × 10^−4^ bar^−1^. The CG simulations were carried out for a duration of 500 ns and the data were saved every 20 ps.

### 3.3. Free Energy Calculations

The Gibbs free energy changes were calculated by using the thermodynamic integration (TI) approach [46] for the alchemical transformation relying on the mutation of a given amino-acid residue. To perform such a transformation all bonded and nonbonded interactions involving the given residue were scaled to an appropriate value in a stepwise manner as a function of a coupling parameter λ. The associated free energy changes were calculated with the Bennett acceptance ratio (BAR) method [47], implemented in the GROMACS gmx bar subroutine, including the error estimation determined by using the default criteria. The 21 evenly spaced λ-points were accepted and the data from equilibrated systems were collected either every 0.1 ps for a duration of 20 ns (all-atom simulations) or every 0.2 ps for a duration of 400 ns (coarse-grained simulations) in each λ window.

The Coulomb and van der Waals parameters were perturbed simultaneously, and a soft-core function was used for the van der Waals interactions to prevent energy singularities. The convergence of the free energy changes was checked by hand-written scripts determining the dependence of this value on the simulation progress.

### 3.4. Pulling Simulations

The pulling simulations concerned with the β2-AR-Gs complex was considered at atomistic resolution. The initial structures were chosen by randomly selecting 24 frames from the equilibrated part of unbiased MD trajectory (in the range of 90–100 ns). The pulling relied on the forced dissociation of the complex by applying external force in a direction perpendicular to the plane of lipid bilayer. During the pulling simulations the force was applied to the center of masses of both β2-AR and Gs molecules to obtain the constant velocity (varying between 0.15 nm/ns and 0.5 nm/ns) of complex dissociation. The value for the corresponding force constant was 5000 kJ/mol/nm^2^. In order to prevent the spurious deformations of the protein structure and the pulling out of the receptor from the lipid bilayer, the position restraints on the protein backbone atoms were applied (100 kJ/mol/nm^2^). In the case of Gs protein, a series of distance constraints were also applied to selected pairs of backbone atoms to keep the secondary structure undisturbed (500 kJ/mol/nm^2^). The free energy of complex dissociation (ΔG, equal to the binding free energy with reversed sign) was calculated from the pulling work using the Jarzynski equality [48,49]:

(1) $$e^{-\beta ∆G}=\langle e^{-\beta W_{i}}\rangle$$

where *β* = 1/*k_B_T* and *W_i_* is the work of each pulling trajectory. The final value was obtained as the average of 24 trajectories of the duration, varying from 8 to 33 ns each.

## 4. Summary and Conclusions

We have investigated the possible influence of a series of natural polymorphisms of the β2-adrenergic receptor structurally located in the transmembrane region on the selected structural and energetic characteristics. The study relied on both atomistic and coarse-grained simulations performed in the presence of explicit solvent.

It has been found that polymorphisms at positions 34, 164 or 220 affect the strength of the interactions of the β2-AR molecule with lipid bilayer only to a very minor extent. Depending on the bilayer composition, the alterations of the associated free energy changes vary between −4.7–+1.3 kJ/mol, as determined by coarse-grained molecular dynamics simulations. Polymorphism at position 220 is located very close to the biding site for the Gs protein; therefore, it was also considered in this context. The associated free energy alteration varies between −0.9–+0.7 kJ/mol (in the case of coarse-grained simulations, values dependent on the bilayer compositions) or is equal 3.5 kJ/mol (for atomistic simulations). The significance of such differences has been determined by estimating the strength of the β2-AR-coupling, expressed as the protein–protein binding free energy and calculated by using atomistic simulations and nonequilibrium pulling methodology. The obtained value of 787 kJ/mol highly exceeds any Ser → Cys-related perturbations. Thus, it can be concluded that, from the perspective of involved energies, this type of polymorphism is not essential for β2-AR-binding.

Apart from free energy calculations, we also determined that some structural descriptors provide insight into the ‘local’ conformational environment of polymorphic amino-acid residues. In the case of the two transmembrane-located polymorphs, the interatomic distances remain weakly affected by the polymorphic transformations. Neglecting some divergences between coarse-grained and atomistic simulations, it can be stated that neither the Val34Met nor Thr164Ile polymorphism is correlated with any notable structural rearrangements in the vicinity of the corresponding amino-acid residues. However, some non-negligible structural alterations, induced by the Ser220Cys polymorphism, have been found in the intracellular region of the receptor (ICL1, ICL2 and ICL3), being the interface for the contact with Gs protein. A series of molecular switches acting through Cys220, Val216, Gln224, Lys227 and Glu268 and leading to conformational rearrangements within the intracellular part of ICL3 and TM6 has been identified. The rearrangements can propagate to ICL2 and ICL1 through interactions mediated by, e.g., Glu225 and Tyr141. All these conformational movements are essentially absent in the case of β2-AR^Ser220^; thus, they can be ascribed to the polymorphism-induced consequences. Judging from the RMSD comparison with the β2-AR-Gs complex, the β2-AR^Ser220^ polymorph structure is more compatible with the Gs-bound receptor. The above findings are qualitatively independent to the type of approach used to treat the missing fragments in the receptor structure, in particular the unstructured ICL3 (‘clipped’ loop, ‘open loop’ and fully reconstructed loop models were tested). Apart from that, we identified the altered solvation around residue 220; the Ser → Cys transformation is correlated with a gain of two or four additional water molecules around the sidechain of Cys.

The current results indicate that the polymorphism-dependent functionality of the β2-adrenergic receptor is not correlated with any simple energetic characteristics, including, e.g., the distinct changes in the affinity to the Gs protein. On the other hand, the Ser220Cys polymorphism-induced alterations of the local protein conformation have sufficient magnitude to be considered in terms of the structural basis of the considered phenomena. Thus, a relatively complex character of the source of polymorphism-related effects can be postulated. Its exact nature and the possible implications for the binding of ligand and G proteins as well as signaling are planned to be investigated in the near future.

## Figures and Tables

**Table 1:** The relative changes of free energies (expressed in kJ/mol) corresponding to mutations of selected amino-acid residues in the molecule of β2-AR. The contributing free energies are associated with the influence of polymorphism on the process of either immersing the receptor into lipid bilayer of different composition (columns 2–5) or of binding the Gs protein by a receptor immersed in the given type of bilayer (columns 6–9, considered only in the context of the Ser220Cys polymorph). Further details in the text.

**CG Simulations (MARTINI)**								
	**Interactions with Lipid Bilayer**				**Gs Binding**			
**Polymorph**	**DPPC**	**POPC**	**POPE**	**Mammalian Bilayer**	**DPPC**	**POPC**	**POPE**	**Mammalian Bilayer**
Val34Met	−0.1 ± 2.4	0.5 ± 1.8	1.3 ± 2.0	−1.8 ± 1.6	-	-	-	-
Thr164Ile	−0.8 ± 2.1	1.0 ± 1.6	3.7 ± 1.3	−4.7 ± 1.7	-	-	-	-
Ser220Cys	0.2 ± 0.1	0.3 ± 0.1	−0.9 ± 0.3	0.1 ± 0.2	0.7 ± 0.1	0.2 ± 0.1	0.4 ± 0.2	−0.9 ± 0.2
**AA Simulations (CHARMM)**								
	**Interactions with Lipid Bilayer**				**Gs Binding**			
**Polymorph**	**DPPC**	**POPC**	**POPE**	**Mammalian Bilayer**	**DPPC**	**POPC**	**POPE**	**Mammalian Bilayer**
Ser220Cys	-	-	-	-	3.5 ± 1.1	-	-	-

## Data Availability

The data that support the findings of this study are available from the corresponding author upon reasonable request.

## Supplementary Materials

The following supporting information can be downloaded at: https://www.mdpi.com/article/10.3390/ijms23105449/s1.
