# Supplementary material for: The Val34Met, Thr164Ile and Ser220Cys Polymorphisms of the β2-Adrenergic Receptor and Their Consequences on the Receptor Conformational Features: A Molecular Dynamics Simulation Study
Source: Int J Mol Sci. 2022 May 13;23(10):5449. doi: 10.3390/ijms23105449 (PMC9141972; doi:10.3390/ijms23105449)
Supplement: Supplementary file 1 [file ijms-23-05449-s001.zip › ijms-1670151-supplementary.pdf]

Supporting Information

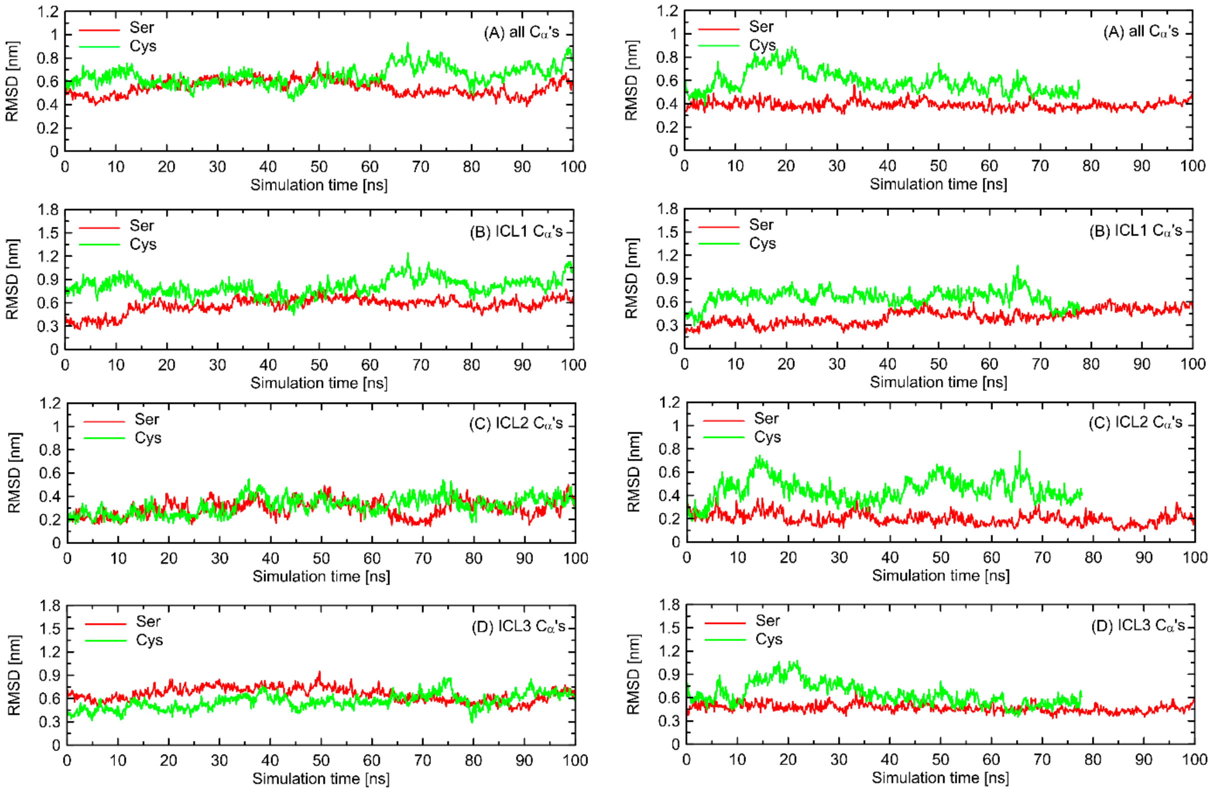

Figure S1. The values of the root-mean square deviation (RMSD) parameters calculated for the β2-AR^{Ser220} and β2-AR^{Cys220} polymorphs with respect to the structure of Gs-bound β2-AR (PDB:3SN6). The data concern the all-atom molecular dynamics (MD) simulations of Gs-free β2-AR with a clipped 3rd intracellular loop (ICL3) model and are the result of the two further, independent MD runs, in comparison to the data illustrated in Fig. 7 (main manuscript). (A) RMSD calculated on the all Cα carbon atoms in all three intracellular loops. (B-D) RMSD calculated on the C_{α} carbon atoms belonging to the particular intracellular loops: (B): ICL1 (Lys60-Thr66); (C) ICL2 (Phe133-Asn148); (D) ICL3 (Ser/Cys220-Glu268).

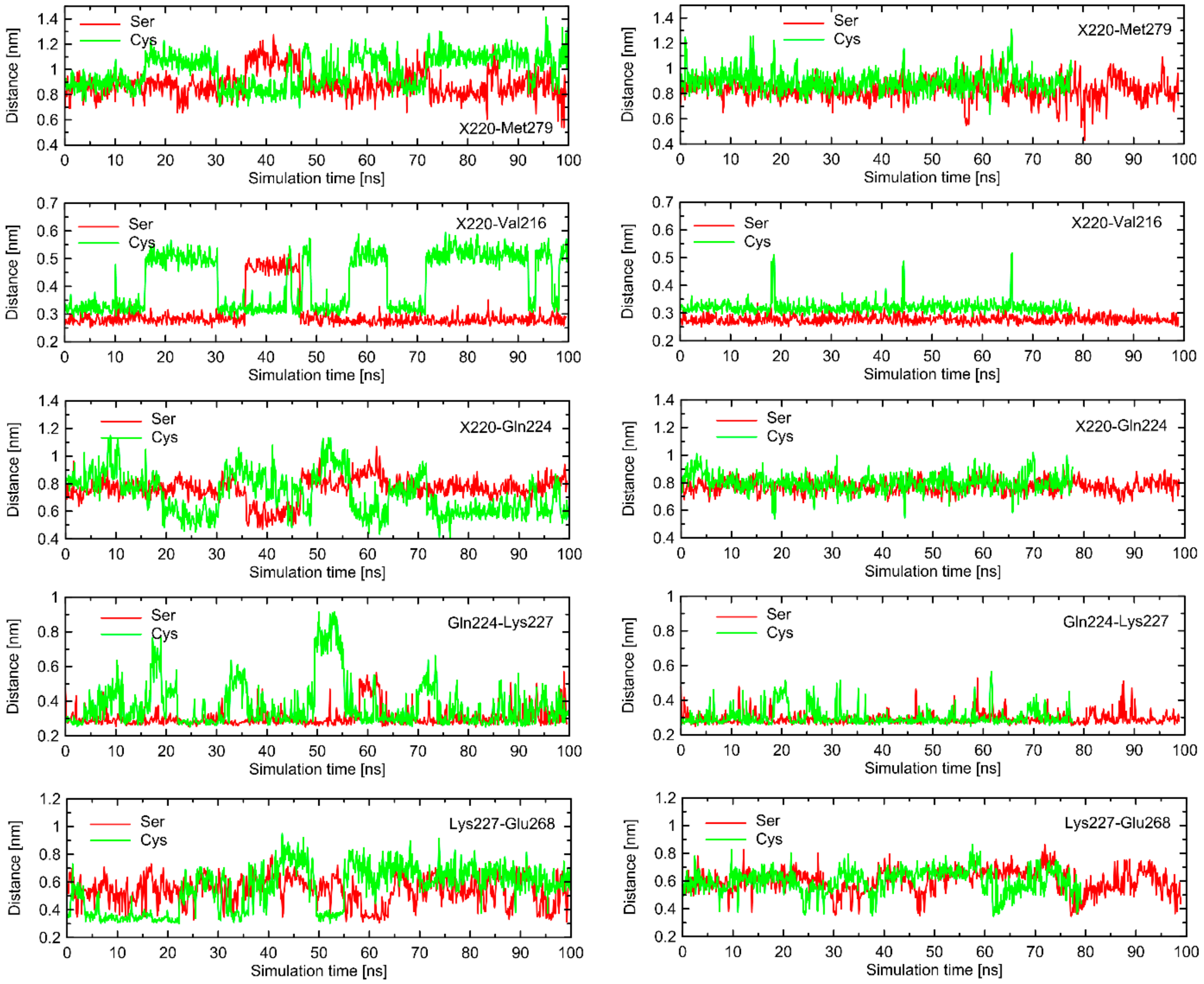

Figure S2. The selected, time-dependent interatomic distances (defined as described in the Methods section) identified in the analysis of MD simulations as potentially important for polymorphism-related effects in G-protein binding by β2-AR. The data concern the all-atom molecular dynamics (MD) simulations of Gs-free β2-AR with the clipped 3rd intracellular loop (ICL3) model and are the result of the two further, independent MD runs, in comparison to the data illustrated in Fig. 8 (main manuscript). The shown distances correspond to structural rearrangements occurring within ICL3. X = Ser or Cys.

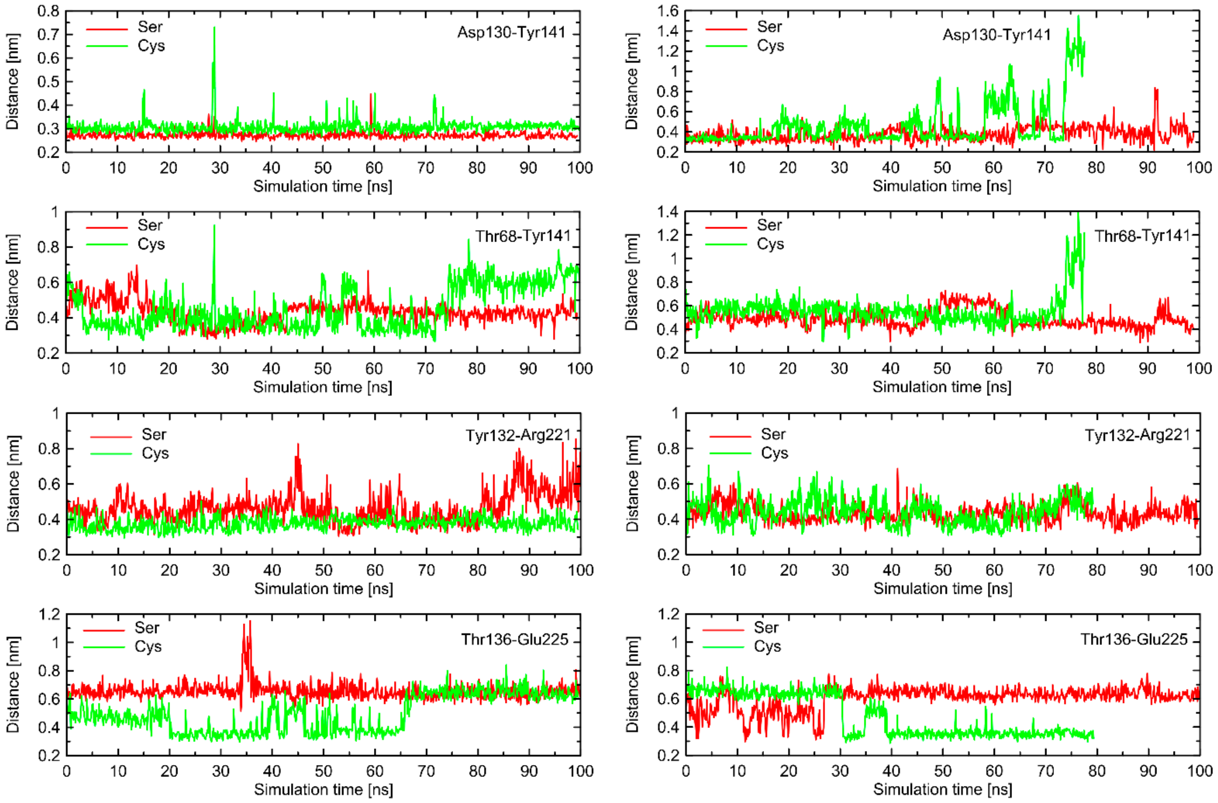

Figure S3. The selected, time-dependent interatomic distances (defined as described in the Methods section) identified in the analysis of molecular dynamics (MD) simulations as potentially important for polymorphism-related effects in G-protein binding by β2-AR. The data concern the all-atom MD simulations of Gs-free β2-AR with the clipped 3rd intracellular loop (ICL3) model and are the result of the two further, independent MD runs, in comparison to the data illustrated in Fig. 9 (main manuscript). The shown distances correspond to structural rearrangements occurring within ICL1 and ICL2. X = Ser or Cys.

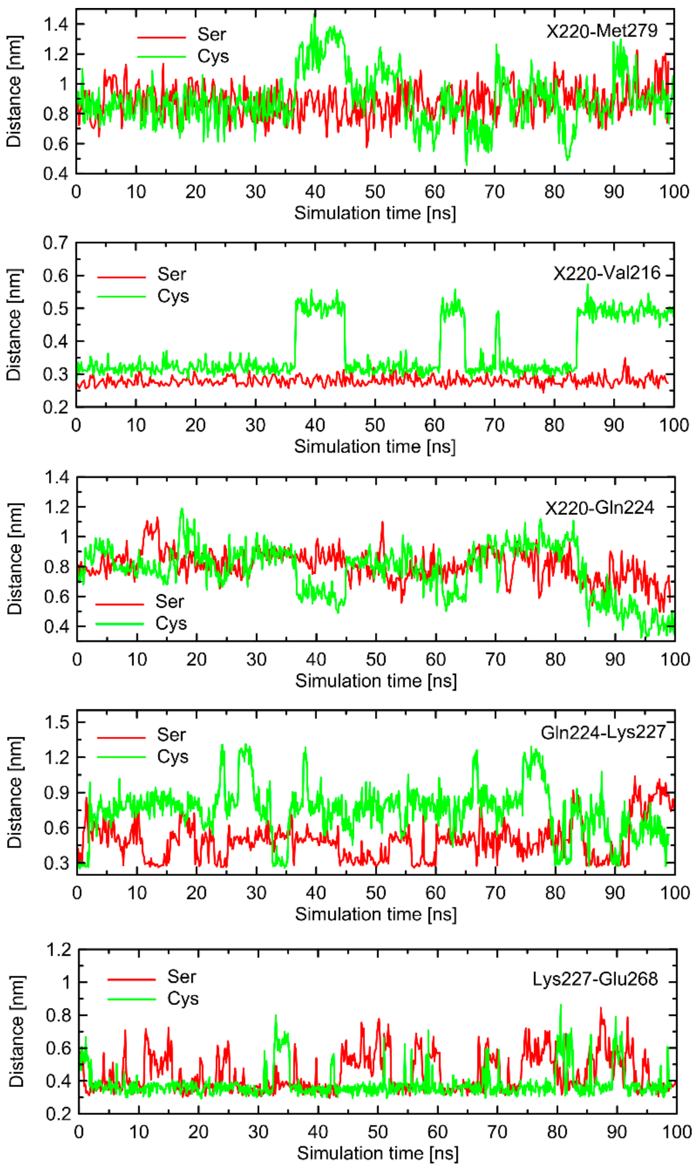

Figure S4. The selected, time-dependent interatomic distances (defined as described in the Methods section) identified in the analysis of molecular dynamics (MD) simulations as potentially important for polymorphism-related effects in G-protein binding by β2-AR. The data concern the all-atom MD simulations of Gs-free β2-AR with a fully reconstructed the 3rd intracellular loop (ICL3) model and are the result of the two further, independent MD runs, in comparison to the data illustrated in Fig. 8 (main manuscript). The shown distances correspond to structural rearrangements occurring within ICL3. X = Ser or Cys.

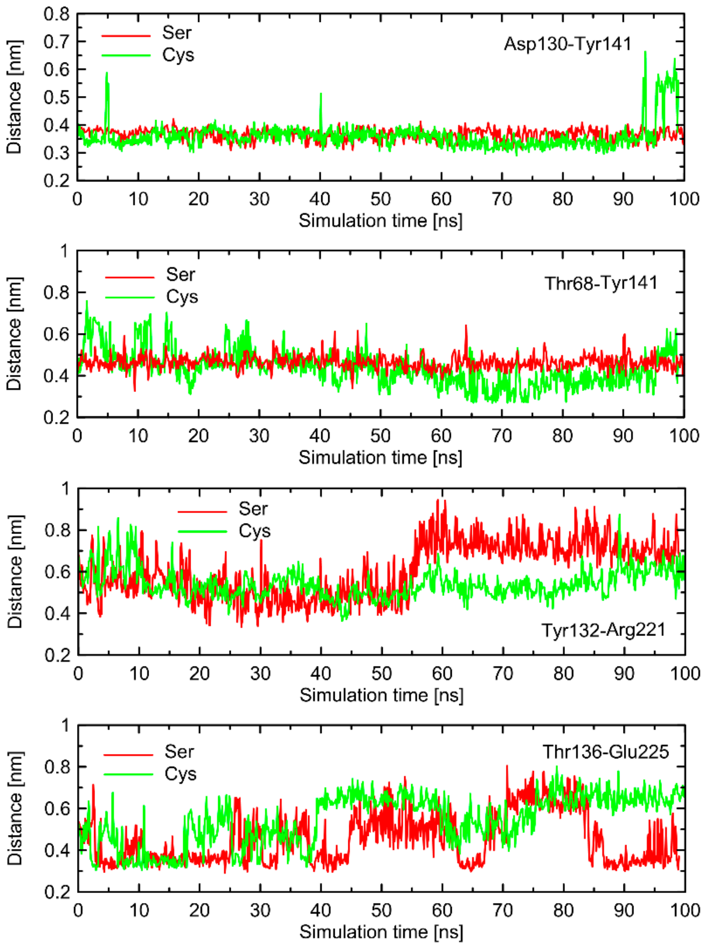

Figure S5. The selected, time-dependent interatomic distances (defined as described in the Methods section) identified in the analysis of molecular dynamics (MD) simulations as potentially important for polymorphism-related effects in G-protein binding by β2-AR. The data concern the all-atom MD simulations of Gs-free β2-AR with a fully reconstructed the 3rd intracellular loop (ICL3) model and are the result of the two further, independent MD runs, in comparison to the data illustrated in Fig. 9 (main manuscript). The shown distances correspond to structural rearrangements occurring within ICL1 and ICL2. X = Ser or Cys.

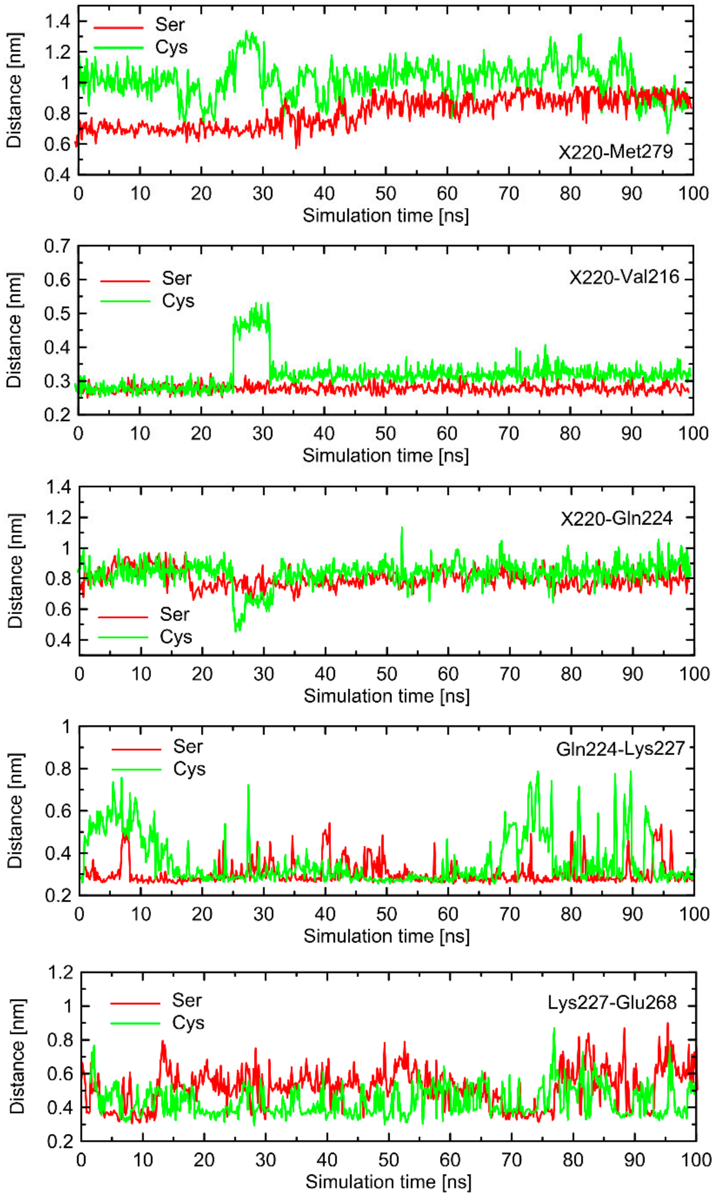

Figure S6. The selected, time-dependent interatomic distances (defined as described in the Methods section) identified in the analysis of molecular dynamics (MD) simulations as potentially important for polymorphism-related effects in G-protein binding by β2-AR. The data concern the all-atom MD simulations of Gs-free β2-AR with an open loop model of the 3rd intracellular loop (ICL3) and are the result of the two further, independent MD runs, in comparison to the data illustrated in Fig. 8 (main manuscript). The shown distances correspond to structural rearrangements occurring within ICL3. X = Ser or Cys.

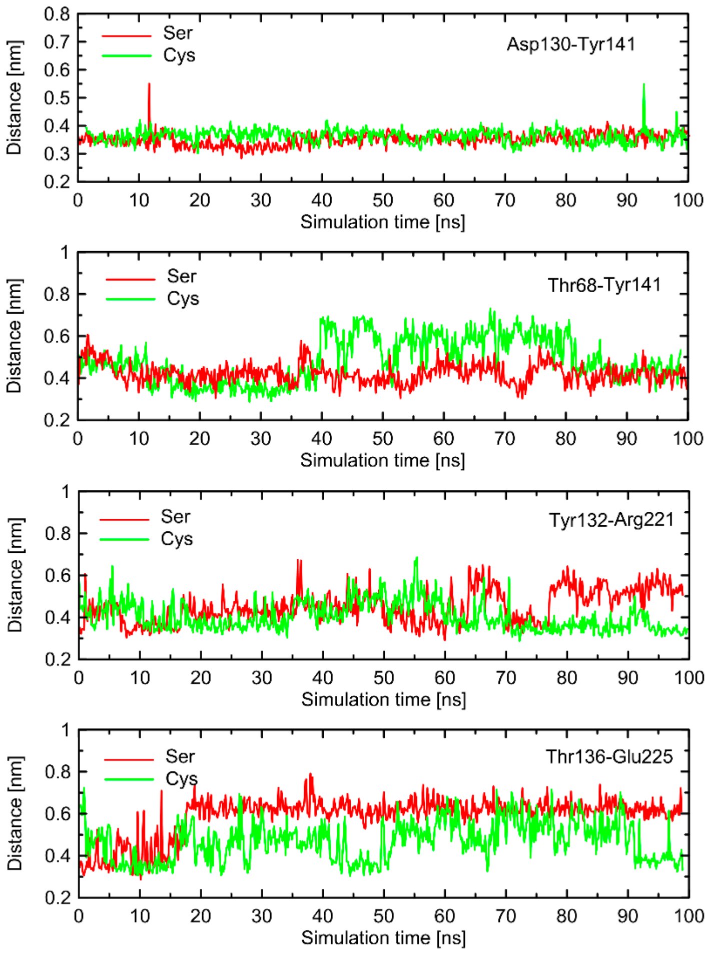

Figure S7. The selected, time-dependent interatomic distances (defined as described in the Methods section) identified in the analysis of molecular dynamics (MD) simulations as potentially important for polymorphism-related effects in G-protein binding by β2-AR. The data concern the all-atom MD simulations of Gs-free β2-AR with an open loop model of the 3rd intracellular loop (ICL3) and are the result of the two further, independent MD runs, in comparison to the data illustrated in Fig. 9 (main manuscript). The shown distances correspond to structural rearrangements occurring within ICL1 and ICL2. X = Ser or Cys.

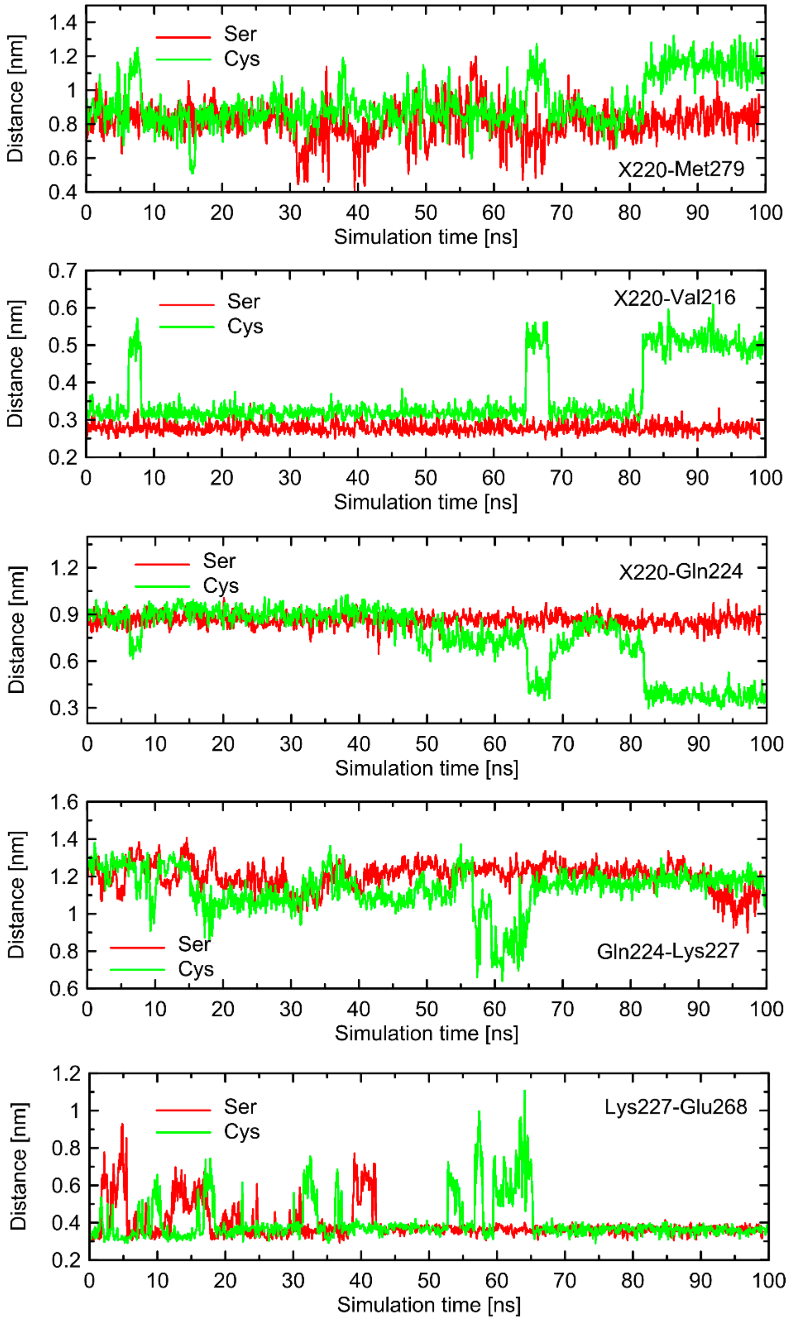

Figure S8. The selected, time-dependent interatomic distances identified in the analysis of molecular dynamics (MD) simulations as potentially important for polymorphism-related effects in G-protein binding by β2-AR. The data were obtained from the unbiased all-atom MD simulations of the β2-AR in complex with Gs (100 ns run, clipped model of the 3rd intracellular loop, ICL3).. The shown distances correspond to structural rearrangements occurring within ICL3. X = Ser or Cys.

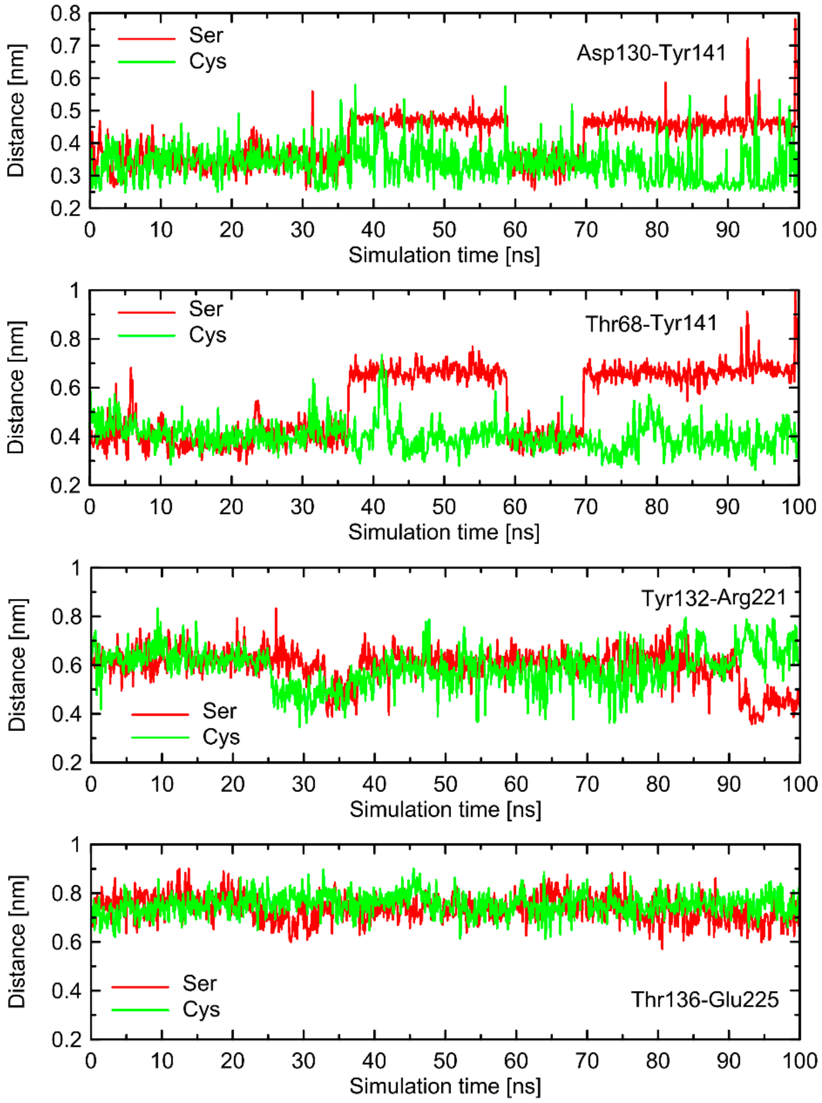

Figure S9. The selected, time-dependent interatomic distances identified in the analysis of molecular dynamics (MD) simulations as potentially important for polymorphism-related effects in G-protein binding by β2-AR. The data were obtained from the unbiased all-atom MD simulations of the β2-AR in complex with Gs (100 ns run, clipped model of the 3rd intracellular loop, ICL3). The shown distances correspond to structural rearrangements occurring within ICL1 and ICL2.
